# Prolonged Unilateral Disuse Osteopenia 14 Years Post External Fixator Removal: A Case History and Critical Review

**DOI:** 10.1155/2010/629020

**Published:** 2010-04-21

**Authors:** Karen M. Knapp, Ann V. Rowlands, Joanne R. Welsman, Kenneth M. MacLeod

**Affiliations:** ^1^College of Engineering, Mathematics and Physical Sciences, University of Exeter, Exeter, Devon EX4 4QL, UK; ^2^School of Sport and Health Sciences, University of Exeter, EX1 2LU Devon, UK; ^3^Peninsula Medical School, Peninsula College for Medicine and Dentistry, EX2 5AX Devon, UK

## Abstract

Disuse osteopenia is a complication of immobilisation, with reversal generally noted upon remobilisation. This case report focuses on a patient who was seen 18 years following a road traffic collision when multiple fractures were sustained. The patient had an external fixator fitted for a tibia and fibula fracture, which remained in situ for a period of 4 years. Following removal, the patient was mobilised but, still required a single crutch to aid walking. Fourteen years post removal of the fixator, the patient had a DXA scan which, demonstrated a T-score 2.5 SD lower on the affected hip. This places the patient at an increased risk of hip fracture on this side, which requires monitoring. There appear to be no current studies investigating prolonged disuse-osteopenia in patients following removal of long-term external fixators. Further research is required to quantify unilateral long-term effects to bone health and fracture risk in this population.

## 1. Introduction

Disuse osteopenia or osteoporosis is a well-recognised complication of immobilisation [1–4] and in the majority of patients there is reversal of the disuse osteopenia upon remobilisation [5]. However, stress fractures distal to the acute fractures have been reported in a small minority of patients post lower limb fracture upon mobilisation [6]. Low trauma fractures have been reported in the lower-limb long-bones of paraplegics [7, 8] and in nonambulatory children with congenital conditions [9], demonstrating that disuse osteopenia results in an increased fracture rate. However, there appear to be no studies at present reporting the long-term effects of prolonged immobilisation of patients placed in external fixators following severe lower limb long-bone fractures. This case report follows a patient who had a dual energy X-ray absorptiometry (DXA) scan 14 years post removal of her external fixator.

## 2. Case Presentation

Eighteen years prior to being seen (1989) the patient had been involved in a major road traffic collision, when she sustained multiple fractures throughout the left side of her body. The patient was aged 19 years at the time of the accident. The collision resulted in fractures of her left radius and ulna, left femur and left tibia and fibula, with open reduction and internal fixation being required for the radius and ulna and femur fractures, with the femoral plate still remaining in-situ. The fracture to the tibia and fibula was a particularly severe compound fracture and an orthofix fixator (Orthofix, TX, USA) was used initially for approximately six months followed by a Sequoia frame (ATS, Paris, France) external fixator to aid healing. Such circular ring fixators can be tolerated for prolonged periods when compared to threaded uniplanar or multiplanar fixators and are therefore a viable option for extended use [10]. The Sequoia ring fixator remained in situ for an extended period of four years, to allow for sufficient new bone growth to bridge the gap left by the fracture. Mobilisation was intermittent during the 4 years over which the patient wore the Sequoia frame external fixator and was dependent upon the patient's pain levels and her ability to weight-bear. Weight-bearing commenced two days post application of the Sequoia frame, with the assistance of two walking sticks, which were changed to crutches since the patient found them too unsteady. Subsequent weight-bearing was impeded intermittently as a result of pain due to infected pin sites, frame adjustment and a corticotomy. Day to day adjustment of the frame was undertaken by the patient. A corticotomy was performed to allow the lower leg to be compressed and new bone to form from the top section of the tibia. A fibulectomy was also performed to allow for free movement of the tibia within the frame, thus allowing stimulation of bone growth to unite the fracture site. Following removal of the external fixator, the patient was mobilised, although even now she uses the assistance of one crutch when walking. The patient underwent extensive physiotherapy, hyperbaric oxygen therapy and manual lymphatic bandaging and massage post removal of the Sequoia frame. Post rehabilitation, while there is no significant limb length discrepancy, the patient suffers from a malunion deformity, which is likely to result in the need for a stick or crutch. 

The patient is however able to fully weight-bear on the affected leg for short periods. During mobilisation, no insufficiency fractures were suffered by the patient. The patient, now 37 years of age, has been without the external fixator for 14 years and an osteoporosis risk factor questionnaire indicated that she currently has none of the standard clinical risk factors for osteoporosis. However, she did undergo a hysterectomy for menorrhagia aged 35 and while still premenopausal at the time of her visit, should premature ovarian failure follow her hysterectomy, this is likely to have a detrimental impact on her bones in the future. The patient is otherwise fit and well, with no history of chronic disease.

## 3. Scans Conducted and Methodology

**Figure 1:**
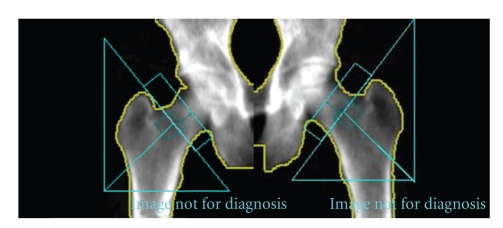
Left and right hip dual energy X-ray absorptiometry scans showing regions of interest used for analysis.

**Figure 2:**
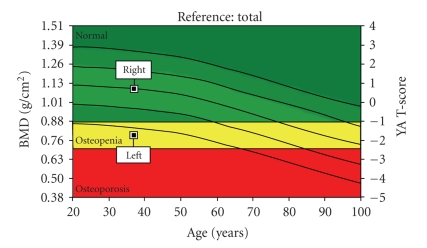
Plot of the BMD against NHANES III reference data for the left and right hips, demonstrating the marked difference between the two.

A dual X-ray absorptiometry (DXA) scan (GE Lunar Prodigy) was conducted of the patients lumbar spine (L1–L4), bilateral proximal femora (Figure 1), and total body. Manufacturers' reference data were used for analysis of the spine and total body results, whilst NHANES III reference data were used for the proximal femora (Figure 2). The results of these scans are shown in Table 1. 

The difference in lean tissue was investigated for the left and right legs using the total body results. These demonstrate similar lean tissue mass on both legs. Using a cohort of 37 normal control subjects, the agreement between the left and right legs for BMD and lean tissue were calculated using Bland-Altman analysis and a comparison made with this case. The results of the Bland-Altman analysis are displayed in Table 2, and demonstrate that this case falls within the normal 95% limits for the difference in her lean tissue mass between the left and right legs. However, the marked reduction in BMD in her affected leg falls well outside the expected 95% limits. 

The hip remains anatomically normal and other reasons for the marked osteopenia such as bone tumours have been excluded.

## 4. Discussion

The disuse osteopenia in this patient is particularly marked. This may be due to a number of factors. Firstly, the patient had an external fixator in situ for an extended period of four years. Secondly, whilst the patient has been mobilised and walks, a crutch is used for assistance, suggesting that the affected leg is not fully weight-bearing at all times. Lastly, the period of partial immobilisation was from age 19–23, an important period for bone accrual and development of peak bone mass [11]. 

There are few studies investigating disuse osteopenia in single limbs. Tandon et al. [12] reported reduced disuse osteopenia following external fixation of the tibia compared to those placed in plaster of Paris, even though those who underwent internal fixation had more severe fractures. Marchetti et al. [13] reported disuse osteopenia following shoulder surgery, which was partially reversed six weeks following remobilisation, whilst Rüegsegger et al. [14] reported bone loss bilaterally post total hip replacement. One of the most frequently studied groups suffering disuse osteoporosis are astronauts following time spent in microgravity during space missions. Lang [15] reported that up to 15% of bone strength can be lost at the proximal femur over a flight of 6 months. Rapid and severe bone loss has been reported in patients suffering stroke [16] and in volunteers on bed-rest studies [17].

Studies of bed-rest volunteers and spinal cord injury patients have consistently reported an increase in markers of global bone resorption [16] Maïmoun et al. [18]. However, in most studies the markers of bone formation have remained unchanged, suggesting that there is no decrease in bone formation as a result of disuse osteopenia [17, 18]. Maïmoun et al. studied the effects of disuse osteopenia on osteoprotegerin (OPG) and reported that OPG was stimulated in spinal cord injury (SCI) patients, whilst nuclear factor *κ*B ligand (RANKL) was inhibited. These results led them to hypothesise that OPG may provide a protective mechanism in the body. Whilst the OPG was deemed to have a protective role in this study, patients still lost bone and bone resorption markers were elevated, suggesting that the stimulation of OPG is insufficient to prevent osteoclastic proliferation and bone resorption [18]. Studies of bed-rest volunteers have also reported increased urinary and faecal excretion of calcium coupled with increased serum calcium and decreased intestinal calcium absorption. Increased serum calcium results in low parathyroid hormone, a regulatory response to the increased bone resorption, which results in a decreased intestinal calcium absorption through the vitamin D mediated pathway [17, 19–21]. 

Nutritional interventions have been reported to have a small influence of addressing the negative calcium balance in disuse osteopenia [22], and early remobilisation is the most important factor for the prevention of disuse osteopenia [7]. However, in patients where this is not possible, other therapeutic interventions may be required. The bisphosphonate Tiludronate has been demonstrated to be an effective treatment for disuse osteoporosis in paraplegic patients [23], whilst aldendronate has been demonstrated to be well tolerated and effective in non-ambulatory children [9]. In an animal study, Ma et al. [24] reported increases in trabecular bone in the tibiae of rats with continuously immobilised hind legs treated with 1,38 human parathyroid hormone (hPTH), suggesting this could be an effective treatment for disuse osteopenia. It is possible that non-pharmacological therapeutic interventions might improve disuse osteopenia such as weight-bearing exercise, or vibrating plates, both of which have been demonstrated to have positive effects on bone density [25, 26].

In conclusion, the current research available on disuse osteopenia, particularly long-term unilateral disuse osteopenia as seen in the patient discussed here is limited. Correct diagnosis means this patient can be monitored and treated, reducing her future fracture risk. Most research is focused on SCI stroke patients, astronauts and bed-rest volunteers and may not be directly comparable to the effects of immobilisation of a single limb. Further research is required to investigate long-term unilateral disuse osteopenia in a wider population, including the fracture prevalence, and possible therapeutic interventions to provide a reduction in their long-term low trauma fracture risk. This is an important consideration for all healthcare teams caring for patients with long-term limb immobilisation or those with only partial remobilisation. The long-term future fracture risk on the affected side and appropriate therapeutic intervention requires consideration.

## Figures and Tables

**Table 1:** The results of the DXA scan are outlined below. Note the 2.5 SD difference between the left and right hip based on the T-score difference (2.4 SD difference on the Z-score).

Site	BMD g/cm^2^	T-score	Z-score*
Lumbar spine (L1–L4)	1.224	0.4	−0.8
Left total hip	0.786	−1.8	−2.4
Right total hip	1.096	0.7	0.0
Total body	1.021	1.0	−0.6

*The Z-scores are weight adjusted.

**Table 2:** Bland-Altman agreement with 95% limits between the left and right legs for BMD and lean tissue in the case report subject and the control population.

	Subject difference	Population mean bias	95% Limits
BMD agreement (g/cm^2^)	−0.317	−0.004	−0.065–0.057
Lean tissue agreement (g)	−328.50	−73.41	−418.73–271.90
